# Wearable Technologies for Diabetic Foot Ulcer Monitoring and Risk Prediction: Systematic Review

**DOI:** 10.2196/84463

**Published:** 2026-07-13

**Authors:** Houriyeh Ehtemam, Simin Salehinejad, Dan Robbins, Alireza Sanaei, Hassan Shirvani, Shabnam Sadeghi-Esfahlani

**Affiliations:** 1 School of Engineering and the Built Environment, Faculty of Science and Engineering, Anglia Ruskin University, Essex, Chelmsford, United Kingdom; 2 Endocrinology and Metabolism Research Center, Kerman University of Medical Sciences, Endocrinology and Methabolism Research Center, Jahad Bolvd, Ebnsina Ave, Kerman, Iran, 98 9132978832; 3 Medical Technology Research Centre, School of Allied Health, Faculty of Health, Education, Medicine & Social Care, Anglia Ruskin University, Essex, Chelmsford, United Kingdom

**Keywords:** diabetic foot ulcer, DFU prevention, foot monitoring, wearable technology, smart insole, Internet of things

## Abstract

**Background:**

Wearable technologies, including smart insoles and sensor-equipped footwear, enable continuous monitoring of key foot parameters such as plantar pressure and temperature in individuals at risk of diabetic foot ulcers (DFUs).

**Objective:**

This systematic review aimed to evaluate the technological characteristics and clinical applications of wearable devices for monitoring DFU-related parameters.

**Methods:**

This review was conducted in accordance with PRISMA (Preferred Reporting Items for Systematic Reviews and Meta-Analyses) guidelines. Journal articles, theses, and dissertations evaluating wearable technologies for DFU prevention or monitoring were eligible if they involved human participants and were published in English or Persian. Studies focused on nonwearable or non-foot–based systems were excluded. A comprehensive search was conducted in PubMed, Embase, Web of Science, and Scopus from July 2024 to May 2025. Two reviewers (HE and SS) independently screened studies and extracted data. The methodological quality of included studies was assessed using the Mixed Methods Appraisal Tool (MMAT) 2018. Results were synthesized using descriptive synthesis.

**Results:**

A total of 1088 records were identified, of which 23 studies met the inclusion criteria. The included studies varied in design, sample size, and follow-up duration. Wearable devices included smart insoles, socks, and external sensors, primarily monitoring plantar pressure and temperature. Devices differed in sensor type, placement, number, communication protocols, and data acquisition rates. Participants typically had diabetes, and many had a history of neuropathy or prior DFUs.

**Conclusions:**

Wearable technologies show promise for monitoring DFU risk factors and supporting early detection. However, the evidence base is limited by heterogeneity in study designs, small sample sizes, and short follow-up periods. Further high-quality studies are required to evaluate their potential clinical benefits, long-term outcomes, and role in preventing DFUs and improving patient care.

## Introduction

Globally, diabetes (both type 1 and type 2) affects approximately 463 million individuals. According to the International Diabetes Federation, this figure is projected to rise to 578 million by 2030 and reach 700 million by 2045 [1]. Diabetes is associated with different medical comorbidities over an extended duration. Diabetic foot ulcers (DFUs) are a common and debilitating complication of diabetes mellitus that can lead to significant morbidity and mortality [2]. DFU is a complex, multifactorial condition that often develops from a combination of peripheral vascular disease, neuropathy, immune dysfunction, Charcot’s neuroarthropathy, previous foot ulceration, and osteomyelitis [3,4]. Consequently, DFUs can lead to nerve damage, resulting in reduced pain sensation and leaving patients unaware of injuries or ulcers. The development of complications can start up to 5 years before a diagnosis of diabetes [5,6].

Approximately 5%-7% of individuals with diabetes are estimated to have experienced, or be experiencing foot ulceration at the time of this writing. In Scotland, a reported 2.5% of the diagnosed diabetic population had active foot ulcers in December 2010 [7,8]. Leading the National Health Service (NHS) to allocate around £1 billion (GBP £1=US $1.29 as of December 31, 2017; approximately US $1.29 billion) annually toward addressing foot care needs of individuals with diabetes [9].

These trends highlight the urgent need for improved DFU prevention strategies [10].

Preventing diabetic foot complications involves a multifaceted approach. This includes lifestyle management, optimizing metabolic control by regulating glucose levels, identifying and screening individuals at high risk for DFUs, and providing patient education to promote foot self-examination and foot care knowledge [11]. Patients are educated to routinely inspect their feet for hyperkeratosis (thickening of the skin’s outer layer), fungal infections, skin lesions, and foot deformities [12]. Footwear control is also emphasized, as repeated trauma from tight or poorly fitting shoes can act as a triggering factor [13]. Achieving this goal can be facilitated by developing affordable and effective wearable devices [14,15]. Wearable technology refers to devices designed to be worn as accessories that facilitate user interaction through both physical inputs and nonphysical modalities, such as voice-based commands [16]. A specific subset of these, referred to as in-shoe wearables, includes devices embedded within or inserted into footwear (eg, smart insoles) for monitoring foot-related parameters such as plantar pressure and temperature. In contrast, nearable devices refer to systems that are not worn on the body but are used in close proximity, such as thermal mats or foot scanners used in clinical or home settings [17]. Recent advances in wearable health care technologies have also been highlighted in broader biomedical applications. A recent review of smart wearable orthoses and sensor-integrated biomedical devices emphasized the growing role of intelligent sensing systems in continuous health monitoring, rehabilitation, and personalized care [18]. The application of skin temperature monitoring represents a promising approach to reduce the risk of DFUs [19]. For example, a clinical trial study conducted by Ming et al [20] has highlighted the significance of thermal imaging techniques in detecting early abnormalities in foot temperature patterns associated with DFUs [20]. Plantar pressure devices are used for monitoring diabetic foot-related issues by measuring weight distribution while standing or walking, which is beneficial for monitoring foot health, especially for people with diabetes [21]. Several studies have addressed the usability of these devices; that is, Gupta et al [22] proposed an insole with high-resolution sensing, equipped with 126 sensing nodes [23]. Their study assessed the functionality of this insole in both static and dynamic conditions, with results confirming its usability for predicting diabetic ulceration. Additionally, the assessment of shear stress (a force that acts parallel to a surface) emerges as another valuable metric for monitoring foot conditions, particularly in predicting the occurrence of DFUs [24].

This study aims to systematically review wearable technologies used in DFU monitoring, focusing on their technical characteristics and reported clinical outcomes.

## Methods

### Overview

Following the PRISMA (Preferred Reporting Items for Systematic Reviews and Meta-Analyses) guidelines, we systematically identified, selected, and evaluated relevant studies for our review [25]. No protocol was registered for this review.

### Ethical Considerations

Given the nature of the review analyzing secondary data, no ethics approval was required.

### Research Strategy

We searched the following databases from July 2024 to May 2025: PubMed, Embase, Web of Science, and Scopus for the English language documents, as well as databases including Scientific Information Database (SID) in Persian.

Search terms were combined using appropriate Boolean operators and included subject heading terms/keywords for 2 key themes (Medical Subject Headings [MeSH] terms), adjusted for each database. The 2 main terms were DFUs (eg, “diabetic foot” OR “diabetic foot ulcer,” OR “DFU,” OR “foot modeling”) AND sensors (eg, “wearable device,” OR “wearable computers,” OR “electronic skin,” OR “wearable technology,” OR “wearable electronic device,” OR “fabric sensor,” OR “smart sock,” OR “smart insole,” OR “remote sensing,” OR “flexible sensor”). Following the database searches, additional manual searching was performed. The records obtained through the database search were managed using EndNote X7 (Clarivate).

To define the research question, the PICO (Population, Intervention, Comparison, and Outcome) framework was applied as follows:

- Population (P): individuals with diabetes or at risk of DFUs;
- Intervention (I): wearable technologies for monitoring foot-related parameters (eg, plantar pressure and temperature);
- Comparison (C): variations in study characteristics and device features;
- Outcome (O): reported technical characteristics and clinical outcomes.

Based on this framework, the research question guiding this review was: “What are the key technological characteristics and clinical outcomes of wearable devices used for monitoring diabetic foot ulcers?”

### Inclusion Criteria

The inclusion criteria focused on studies published as journal articles, theses, and dissertations. The following criteria guided study selection: (1) the study evaluated wearable devices, either commercially available or researcher-developed, used for foot monitoring, including in-shoe systems such as smart insoles, sensor-integrated footwear, smart socks, or similar wearable sensor devices that measured parameters such as plantar pressure, temperature, or humidity; (2) published in English or Persian; (3) it involved human participants rather than laboratory animals; and (4) it addressed either the prevention of DFUs (eg, monitoring high-risk individuals with neuropathy or a history of prior ulcers) or the monitoring of active DFUs (eg, wound progression and healing). Studies were excluded if they did not use systems or sensors attached to the inside of the shoe or inserted, such as insoles or other removable devices, to monitor DFUs.

### Study Selection

After removing duplicates, the first and second co-authors (HE and SS) independently screened titles and abstracts to remove irrelevant studies. To confirm eligibility, studies passing the title-abstract screening were examined by the first and second authors (HE and SS) at the full-text screening phase. In the last phase, the full texts of articles were assessed again by the first and second authors (HE and SS) to determine their originality and to extract data. Interrater reliability was assessed during the full-text screening stage using Cohen kappa [26]. All screening phases were conducted independently using EndNote and Excel (Microsoft Corp).

### Data Extraction

Data extraction was completed independently by the first and second authors (HE and SS). When needed, the first and second authors (HE and SS) checked their data extraction process with the supervisor for accuracy and clarifications. Data extraction was conducted using Microsoft Excel 2016 spreadsheet. The following information was extracted from each included study: (1) study characteristics, including authors of the study, year of data publication, study location, study design, and sample size; (2) participant characteristics, including type of diabetes (if mentioned), follow-up period, and history of DFUs and neuropathy; (3) technical characteristics, including application of wearable devices, key metrics, sensors’ location, count of sensors, sensors’ technology, communication method, range of measurement, acquisition rate or sampling frequency, and user interface; and (4) clinical outcomes. When information was not directly available, the corresponding authors (SS) were contacted to obtain additional details.

### Quality Assessment

The quality of the included articles was assessed using the Mixed Methods Appraisal Tool (MMAT 2018) following the search process, which includes a checklist to appraise the methodological quality for qualitative, quantitative, and specifically mixed methods studies. MMAT’s 5 sets of criteria were adopted to evaluate the quality of each type of study included in our analysis, namely qualitative, randomized controlled trial (RCT), nonrandomized, quantitative, descriptive, and mixed methods studies [27]. This rigorous assessment process allowed us to evaluate the included studies’ methodological quality and ensure our findings’ reliability and validity (Table S1 in Multimedia Appendix 1 [3,19,20,22,28-68]).

### Bias Management

To limit selection bias, we did not apply any restrictions regarding the type of study or the population studied, allowing for the inclusion of a broad and diverse range of studies. To reduce publication bias, we excluded previously published review and meta-analysis papers, focusing instead on primary studies to ensure the originality of the included data. In addition to peer-reviewed journal articles, gray literature sources such as theses and dissertations were not excluded (if available) to minimize publication bias and capture a broader range of evidence. These sources should be identified through database searches and screened using the same eligibility criteria as published studies.

The review included studies published in both English and Persian to enhance the comprehensiveness of the search and reduce potential language bias. Although in the final included studies there is no Persian study because of not having inclusion criteria, the investigation of Persian-language studies allowed for making sure about the existing of additional relevant evidence that may otherwise have been overlooked. All included studies were assessed using the same eligibility criteria to ensure consistency. However, studies published in other languages were not searched, which may still introduce some degree of language bias.

To address citation bias, we did not rely solely on highly cited articles or those published in high-impact journals; instead, we conducted a comprehensive and systematic search across multiple databases to capture both widely recognized and less frequently cited studies. Finally, to minimize multiple-publication bias, we carefully reviewed the included studies to identify and exclude duplicate reports or multiple publications from the same dataset, ensuring that each study contributed uniquely to the analysis.

## Results

### Overview

**Figure 1.**
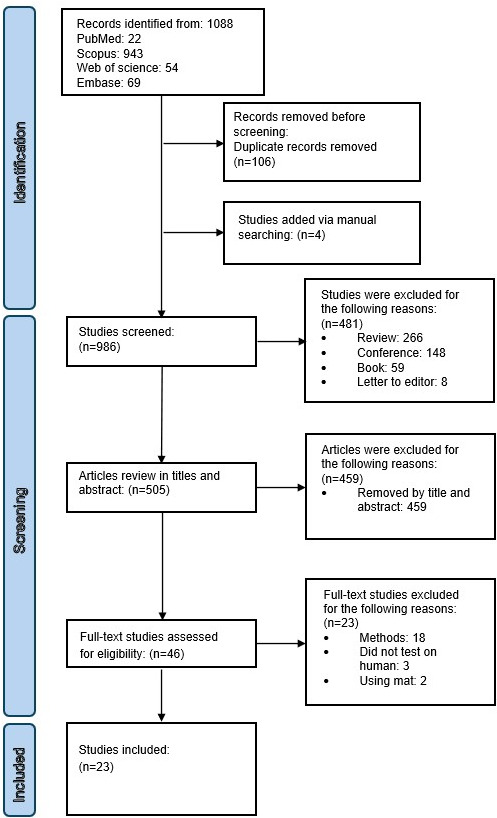
PRISMA (Preferred Reporting Items for Systematic Reviews and Meta-Analyses) flowchart describing the selection of studies for the systematic review.

A total of 23 articles were included in this systematic review (Figure 1), comparing various wearable device technologies used in monitoring DFUs and their technical characteristics and clinical participant-related features (Tables1  2).

**Table 1.** Descriptive summary of articles included in the study (part 1).

No.	Authors, published year	Country	Study design	Sample size	Follow‐up period	Foot ulcer	Type of diabetes	Application type	Monitored metrics	Interface
						Neuropathy				
1	Hochlenert et al (2025) [28]	Germany	Clinical trial	17	7.5 months	Foot ulcer	Type 1 or 2	Bandage	Pressure, temperature, humidity, and steps	Smartwatch
2	Bulathsinghala et al (2024) [29]	Sri Lanka	Cohort	16	25 days	11 participants had foot ulcer	Type 1 or 2	Bandage	Blood flow and temperature	Smartphone
3	Cay et al (2024) [30]	United States	Clinical trial	62	3 months	Foot ulcer	Not reported	Boot	Offloading adherence, daily steps, and cadence	Smartwatch
4	Hu et al (2024) [31]	China	Clinical trial	23	No follow-up	None	Type 2	Anklet	Blood flow	Not reported
5	Park et al (2023) [32]	United States	Case series	14	No follow-up	None	None	Boot	Adherence, gait, and balance	Smartwatch
6	Tang et al (2023) [33]	United Kingdom	Case-control	6	No follow-up	None	Not reported	Insole	Pressure and shear stress	Not reported
7	Gupta et al (2023) [22]	India	Cross-sectional	50	No follow-up	Not reported	Not reported	Insole	Pressure	Not reported
8	Armstrong et al (2023) [34]	United States	Prospective study	10	3 months	Foot ulcer	Not reported	Boot	Pressure	Not reported
9	Khandakar et al (2022) [3]	Qatar	System analysis	12	No follow-up	Not reported	Not reported	Insole	Pressure and temperature	Smartphone
10	Reyzelman et al (2022) [35]	United States	Case-control	31	More than 50 days	Neuropathy	Type 1 or 2	Sock	Temperature	—^a^
11	Scholten et al (2022) [36]	United States	Cross-sectional	147	7 months	Neuropathy	Not reported	Sock	Temperature	—
12	Samarentsis et al (2022) [37]	Greece	Development evaluation	1	No follow-up	None	None	Insole	Pressure	PC^b^
13	Beach et al (2021) [38]	United Kingdom	Clinical trial	25	No follow-up	None	Type 1 or 2	Insole	Temperature	Smartphone
14	Du et al (2021) [39]	China	Clinical trial	6	6 months	Neuropathy	Type 1	Sensor	Gait and balance	Not reported
15	Torreblanca González et al (2021) [19]	Spain	Clinical trial	93	No follow-up	Not reported	Not reported	Sock	Temperature	Smartphone
16	Abbott et al (2019) [40]	United Kingdom	Clinical trial	58	18 months	Both	Type 1 or 2	Insole	Pressure	Smartwatch
17	Huchegowda et al (2019) [41]	India	Cross-sectional	30	No follow-up	Neuropathy	Type 2	Insole	Pressure	Smartphone
18	Ming et al (2019) [20]	Germany	Clinical trial	300	24 months	Neuropathy and third group had history of foot ulcer	Type 1 or 2	Insole in slippers	Temperature	Smartphone
19	Reyzelman et al (2018) [42]	United States	Observational study	35	7 days	Neuropathy and the second and third groups include foot ulcers and pre-ulcer	Type 1 or 2	Sock	Temperature	Smartphone
20	Zhou et al (2018) [43]	United States	Cross-sectional	196	No follow-up	None	None	Anklet	Gait and balance	PC or tablet
21	Coates et al (2016) [44]	United Kingdom	Cross-sectional	16	No follow-up	None	None	Sensor	Acceleration, rotation, GSR^c^, environmental temperature, humidity, force, skin temperature, and bioimpedance signals	Arduino devices
22	Grewal et al (2015) [45]	Qatar and United States	Clinical trial	39	1 month	Neuropathy	Type 2	Body-worn sensor technology	Balance, daily physical activity, triaxial accelerometer, gyroscope, and magnetometer	PC
23	Wrobel et al (2014) [46]	United States	Cross-sectional	27	No follow-up	Neuropathy	Type 1 or 2	Insole	Temperature, sudomotor function, gait, and balance	Not reported

a Not available

b PC: personal computer.

c GSR: galvanic skin response.

**Table 2.** Descriptive summary of studies included in the study (part 2).

Number	Authors (year)	Sensor location	Sensor count	Sensor type	Connectivity	Measurement range	Sampling and acquisition rates (Hz)
1	Hochlenert et al (2025) [28]	Under the ulcer (variable)	1	Custom built	Bluetooth	Not reported	≈ 0.00167
2	Bulathsinghala et al (2024) [29]	Left and right forefoot areas	2	Not reported	Bluetooth	±2 °F	50
3	Cay et al (2024) [30]	Shin	1 per leg	Balance and gait IMU^a^	BLE^b^	Accelerometer: ±2 g Gyroscope: ±2000 deg/s	≈ 100
4	Hu et al (2024) [31]	Medial forefoot M5^c^ Heel M1	4	Laser doppler flowmetry	Bluetooth	0.8‐4.5 mm/s	20
5	Park et al (2023) [32]	Lower back+upper thighs Shin	5	Balance and gait IMU	BLE	Accelerometer: ±2 g Gyroscope: ±2000 deg/s	≈ 100
6	Tang et al (2023) [33]	MH1^d^ MH5 Hallux	4	Custom built, capacitive	Wireless	Pressure: 0 _ 300 kPa Shear stress: ±90 kPa	100
7	Gupta et al (2023) [22]	Lateral heel Toes Medial forefoot Central forefoot Lateral forefoot Midfoot Heel	126	Piezoresistive	Wi-Fi	5 _ 900 kPa	40
8	Armstrong et al (2023) [34]	—^e^	—	Compression pump	Not reported	—	—
9	Khandakar et al (2022) [3]	Toes Metatarsals Medial and lateral arch Heel	24	Piezoelectric	BLE	Temperature: 20 _ 50 ◦C Pressure: 80 _ 600 kPa	40
10	Reyzelman et al (2022) [35]	M1 M3 M5 Hallux Heel Arch	6	Resistive	—	20 _ 40 °C	≈ 0.1
11	Scholten et al (2022) [36]	M1 M3 M5 Hallux Heel Arch	6	Resistive	—	20 _ 40 °C	≈ 0.1
12	Samarentsis et al (2022) [37]	Metatarsals Toes Lateral arch Heel	16	Capacitive	Bluetooth	41.5 _ 872.4 kPa	Not reported
13	Beach et al (2021) [38]	MH1 MH5 Hallux Heel	4	Resistance	BLE	22 _ 42 ^◦^C	1
14	Du et al (2021) [39]	Gait sensors Bilateral lower shins Balance sensors Lower back Lower left shin	4	Balance and gait IMU	Bluetooth	Accelerometer: ±2 g Gyroscope: ±2000 deg/s	100
15	Torreblanca González et al (2021) [19]	MH1 MH5 Heel Medial and lateral arch Hallux	6	Resistive	Bluetooth	0 _ 43 ^◦^C	≈ 0.1
16	Abbott et al (2019) [40]	MH1 Lateral metatarsal heads Hallux Lateral toes Lateral arch Heel	8	Resistive	Wireless	25 _ 225 mm Hg	≈ 8
17	Huchegowda et al (2019) [41]	Not reported	Not reported	Capacitive	Bluetooth	Not reported	Not reported
18	Ming et al (2019) [20]	MH1 MH3 MH5 Hallux Lateral Heel	6	Capacitive	BLE	15 _ 600 kPa	2
19	Reyzelman et al (2018) [42]	M1 M3 M5 Hallux Midfoot Heel	6	Resistive	Bluetooth	20 _ 40 °C	≈ 0.1
20	Zhou et al (2018) [43]	Left and right lower shins	2	Balance and gait IMU	Bluetooth	Accelerometer: ±2 g Gyroscope: ±2000 deg/s	≈ 100
21	Coates et al (2016) [44]	M1 M5 Heel Hallux	42	Accelerometer: IMU Humidity: capacitive Temperature: not reported GSR:^f^ IMU Bioimpedance: digital frequency synthesis Force: piezoresistive	Bluetooth	Accelerometer: ±16 g Rotation: ±2000 ◦s−1 Humidity: 0%‐99 % RH Temperature: -40‐125 ◦C GSR: a 0‐5000 kΩ Bioimpedance: 0‐1023 AU^g^ Force: 0‐140 N Temperature skin: 20‐40 ◦C	20
22	Grewal et al (2015) [45]	Shin Thigh Lower back	5	Balance and gait IMU	Bluetooth	Accelerometer: ±2 g Gyroscope: ±2000 deg/s	≈ 100
23	Wrobel et al (2014) [46]	Midfoot Forefoot Hindfoot Entire foot	≈ 5	Balance and gait IMU	Bluetooth	Accelerometer: ±2 g Gyroscope: ±2000 deg/s	≈ 100

a IMU: internal measurement unit.

b BLE: Bluetooth low energy.

c M: metatarsal.

d MH: metatarsal head.

e Not applicable.

f GSR: galvanic skin response.

g AU: arbitrary units.

Interrater reliability between the 2 reviewers was assessed using Cohen kappa. The level of agreement was high (κ=0.87; Table S2 in Multimedia Appendix 1).

### Quality Assessment

Most of the included studies were good in terms of quality. Of the 23 included papers, 4 [40,43-45] studies met all MMAT criteria (2 RCTs and 2 nonrandomized controlled trials). In 7 [19,20,28,30,31,38,39] RCTs, assessors blinded to the intervention were not clarified (Table S1 in Multimedia Appendix 1).

### Year-Oriented Analysis

The dataset includes studies published between 2014 and 2025, highlighting a growing academic interest in the field. The distribution of publications indicates an upward trend, with the highest number of studies published in 2022 (n=4) [3,35-37] and 2023 (n=4) [22,32-34], with 2024 and 2019 each n=3 [20,29-31,40,41]. In contrast, the fewest publications were recorded in 2014, 2015, 2016, and 2025, with each year contributing only one study [28,44-46]. A notable gap in research output was observed between 2016 and 2018, during which no studies met the inclusion criteria. In 2020, 2 [47,69] studies used mats; however, since mats are not considered wearable devices, these studies were excluded.

### Country-Based Analysis

The geographical distribution of the reviewed studies shows a strong concentration of research in the United States, where 9 [30,32,34-36,42,43,45,46] studies were conducted. Additionally, 4 studies were conducted in the United Kingdom [33,38,40,44], while China [31,39], Qatar [3,45], India [22,41], and Germany [20,28] each contributed 2 studies. Other studies were conducted in Greece [37], Spain [19], and Sri Lanka [29].

### Study Design Analysis

The reviewed studies used a variety of research designs. Nine [19,20,28,30,31,38-40,45] were identified as clinical trials, while 6 [22,36,41,43,44,46] studies used a cross-sectional design. Two [33,35] studies used a case-control design, and 2 [3,37] used system analysis and development evaluation. One observational [42], one prospective study [34], one case series [32], and one cohort study [29] were also identified.

### Sample Size Analysis

The sample sizes across the reviewed studies showed considerable variation, ranging from as few as a single participant [37] to as many as 300 participants [20]. Despite this wide range, the average sample size was approximately 26 participants.

### Follow-Up Period Analysis

The study with the longest follow-up period tracked participants for 24 months [20]. This clinical trial aimed to assess the effectiveness of telemedicine and foot temperature monitoring in reducing the risk of ulcer formation. Several other studies followed participants for shorter durations, specifically 18 [40], 7 [28,36], 6 [39], and 3 months [30,34]. However, most of the studies did not include a follow-up period and relied on a single laboratory test [3,19,22,31-33,37,38,41,43,44,46].

### Participant Analysis

Nine [20,35,36,39-42,45,46] studies included participants with a history of neuropathy, while 7 [20,28-30,34,40,42] focused on those with either active ulcers or a history of DFUs. The remaining studies either did not specify participants’ neuropathy or ulcer history or confirmed that participants had no such conditions. Eight [20,28,29,35,38,40,42,46] studies included participants with a history of diabetes, either type 1 or 2. Three [31,41,45] studies specifically involved participants with type 2 diabetes, and one [39] study included participants with type 1 diabetes. The remaining studies did not specify the type of diabetes.

### Wearable Device Applications Analysis

Nine [3,20,22,33,37,38,40,41,46] studies developed insoles, while 4 [19,35,36,42] focused on socks, and 3 [30,32,34] on boots. The remaining studies either did not specify the type of application or involved only sensors and bandages.

### Key Metrics Analysis

Eleven [3,19,20,28,29,35,36,38,42,44,46] studies reviewed focused on monitoring temperature. Eight [3,22,33,34,37,40,41,44] studies monitored pressure. In addition to temperature and pressure, several other metrics were considered important for ulcer prediction. Gait and balance were reported in 4 [32,39,43,46] studies, humidity in 2 [28,44] studies, daily step count in 2 [28,30] studies, and blood flow in 2 [29,31] studies. Force [44], shear stress [33], galvanic skin response [44], and bioimpedance signals [44] were each reported in one study.

### User Interface Analysis

Smartphones were used in 7 [3,19,20,29,38,41,42] of the reviewed studies, likely due to their portability and ease of integration with wearable sensor systems. Their built-in computing power and wireless connectivity enabled real-time data processing and supported user-friendly application interfaces.

Personal computers were used in 3 [37,43,45] studies that required more complex computational analysis. These tasks included machine learning–based gait assessment and high-resolution pressure mapping, where greater processing capacity was required to handle large datasets and detailed modeling.

### Technical or Engineering Outcomes

#### Sensors’ Location Analysis

**Figure 2.**
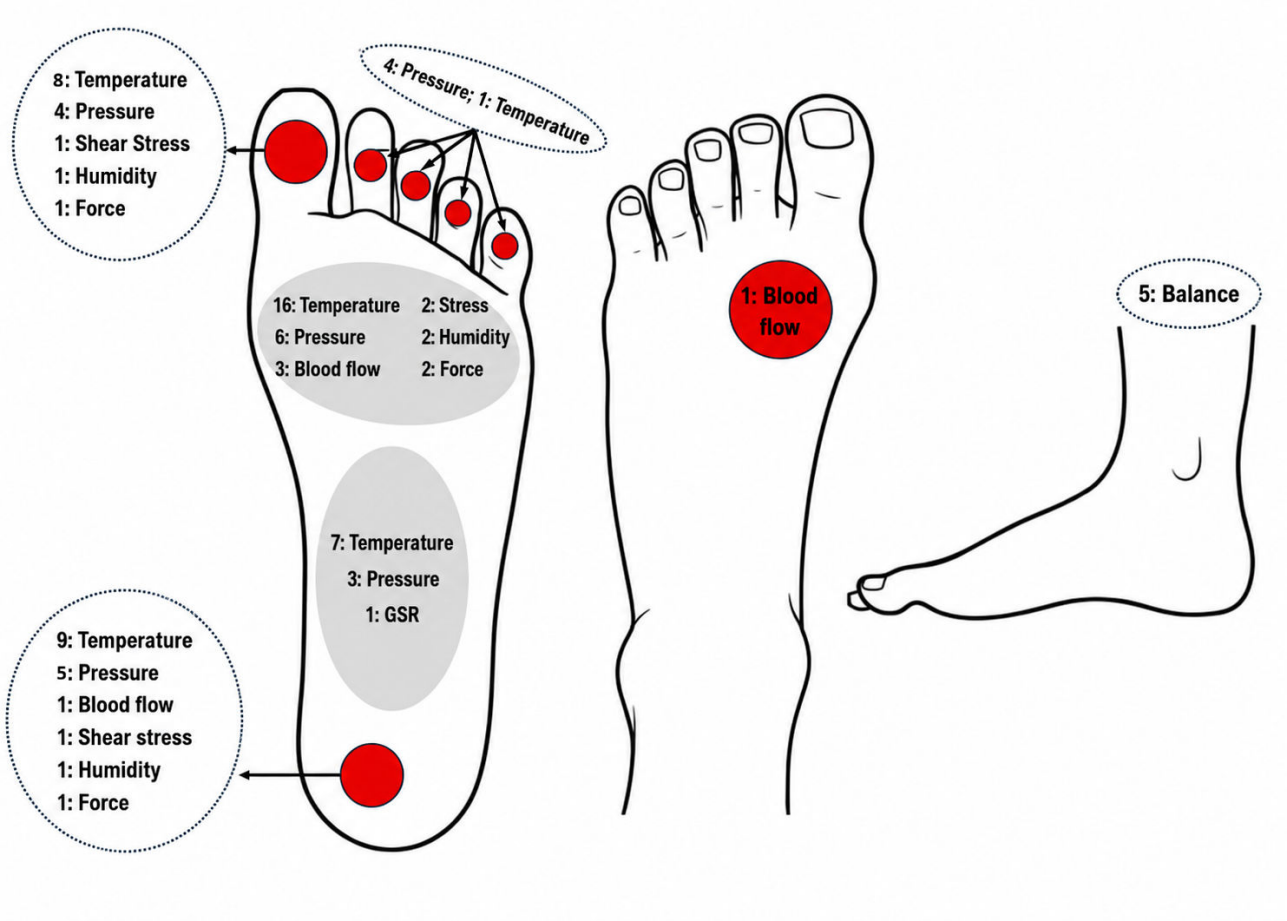
Distribution of studies by sensor location and number of sensors. GSR: galvanic skin response.

The studies used various sensor placement strategies to capture foot-related biomechanical data. Most studies measuring temperature placed sensors on the first, third, and fourth metatarsals, as well as on the heel, arch (medial and lateral), and hallux [19,20,35,36,38,42,44,46]. For pressure measurements, sensors were typically placed on the first metatarsal head, lateral metatarsal heads, hallux, lateral toes, lateral foot, toes, arch (medial and lateral), and heel [22,33,37,40]. Moreover, studies focusing on gait and balance commonly placed sensors on the lower back and dominant lower shin [30,32,39,43-46] (Figure 2).

#### Number of Sensors Analysis

The number of sensors used across the reviewed studies varied considerably, reflecting differences in study objectives, measurement complexity, and technological capabilities. Sensor configurations ranged from single-sensor systems to arrays consisting of 5 [32,45,46], 6 [19,20,35,36,42], 8 [40], 16 [37], 24 [3], or even 42 sensors [44] (Figure 2).

#### Sensors’ Technology Analysis

Various sensor technologies were used, including piezoresistive, capacitive, and piezoelectric sensors. Seven [30,32,39,43-46] studies used a balance and gait internal measurement unit, 6 [19,35,36,38,40,42] studies referenced resistive sensors, 5 [20,33,37,41,44] studies mentioned capacitive sensors, and 3 [3,22,44] studies mentioned using piezoresistive and piezoelectric sensors. The remaining studies used laser Doppler flowmetry [31] or a compression pump [34], while others did not report the sensor technology used in developing their wearable devices.

#### Communication Method Analysis

Generally, 2 main types of communication protocols are used in microcontroller systems: one facilitates communication between the sensors and the microcontroller (eg, Interintegrated Circuit, Serial Peripheral Interface, and Universal Asynchronous Receiver-Transmitter), and the other manages communication between the microcontroller and the reference device or server (eg, Bluetooth and Wi-Fi). The reviewed studies primarily used wireless communication technologies, with Bluetooth and Wi-Fi being the most common. Among the 23 studies analyzed, Bluetooth was the preferred method, used in 12 [19,28,29,31,37,39,41-46] studies. Notably, 5 [3,20,30,32,38] studies specified the use of Bluetooth low energy (BLE), emphasizing energy-efficient data transmission, which is particularly beneficial for wearable sensor applications. In addition to Bluetooth, other general wireless communication technologies were used in 3 studies, although the specific protocols were not always clearly defined [22,33,40].

#### Range of Measurement Analysis

The measurement range varied considerably across the reviewed studies, primarily depending on the type of sensor and its intended application. Specifically, pressure sensors demonstrated a broad detection capability, spanning from 5 kPa to 900 kPa [3,20,22,33,37]. This wide range allows for the assessment of both low-pressure and high-impact forces, making such sensors suitable for applications in gait analysis, foot biomechanics, and clinical diagnostics.

Similarly, temperature sensors were used in several studies, with a reported measurement range of 0 °C to 50 °C [3,19,29,35,36,38,42,44]. This range aligns with physiological temperature variations and external environmental influences, both of which are crucial for monitoring foot health, particularly in patients with diabetes and individuals with circulatory disorders.

#### Acquisition Rate or Sampling Frequency

The sampling frequency of sensor systems varied across the reviewed studies, ranging from 0.001 Hz to 100 Hz. The reported sampling frequency may not reflect the maximum capability of the sensors. Typically, we reported the value presented in the articles; however, it is possible that the sensors can record or acquire data at higher frequencies.

Higher sampling rates are generally associated with improved temporal resolution, enabling more precise capture of dynamic biomechanical events. The highest reported acquisition rate was 100 Hz [30,32,33,39,43,45,46], which facilitates detailed gait analysis by capturing rapid changes in foot pressure and movement patterns. However, higher rates also increase the risk of recording noise [70].

Lower sampling frequencies (eg, 1 Hz) may still be adequate for applications where slower physiological changes are monitored, such as prolonged pressure distribution assessments. However, for real-time gait analysis or high-impact activities, higher acquisition rates are preferable to avoid data loss and improve the accuracy of motion tracking.

Overall, the findings suggest that most current wearable systems are mainly designed for preventive monitoring, particularly through measuring plantar pressure, temperature, and gait patterns. However, considerable variation in sensor choice, communication methods, and reporting standards makes it difficult to directly compare devices across studies. More consistent technical reporting and standardized benchmarking approaches are needed to enable clearer comparisons and support future clinical implementation.

### Clinical Outcomes

Wearable sensors for DFUs in patients with diabetes have demonstrated a broad range of positive clinical outcomes across multiple studies (Table S3 in Multimedia Appendix 1).

These technologies enabled early detection and prevention of foot ulcers and related complications, facilitating timely intervention and treatment [3,19,29,31,35,39,40,42,43,46].

A small subset of studies focused on patients with active DFUs, where wearable technologies were associated with accelerated wound healing, increased wound closure rates, reduction in wound area, and pain relief. For example, 2 [28,34] studies reported that wearable sensors accelerated wound healing and increased wound closure rates, while others showed significant wound area reduction and pain relief [34,41].

### User-Level Outcomes

Wearable sensors demonstrated substantial benefits in the early detection and prevention of DFUs across multiple studies. These technologies enabled timely intervention by continuously monitoring risk factors such as plantar pressure and temperature. In 7 [28,30,35,37,40,42,45] studies, these devices supported patient-centered care by empowering patients to self-monitor, improving engagement, and enabling personalized treatment plans tailored to individual risk factors and healing barriers. Real-time tracking of patients’ adherence to offloading allowed proactive intervention when patients deviated from prescribed care [32]. Remote and at-home monitoring, reported in 5 studies, reduced the need for frequent clinic or hospital visits, making long-term management more accessible [3,19,30,31,35]. Functional improvements such as better gait, balance, postural stability, and overall mobility were described in 5 [32,33,39,41,45] studies. The cost-effectiveness of these technologies was highlighted in 3 [3,29,37] studies. Mental health benefits, including increased confidence and reduced anxiety, were reported in one study [45], while quality of life improvements were noted in another [28]. Furthermore, wearable sensors proved suitable for routine clinical use and long-term monitoring without disrupting patients’ daily activities [19,31].

## Discussion

### Overview of Research Trends and Study Characteristics

As the results indicate, there was no publication between 2016 and 2018 and low publication before 2016; however, the rise in studies since 2019 demonstrates a renewed research interest, likely driven by advances in microcontrollers and miniaturized sensors. Additionally, inclusion studies were conducted in various countries, from the United States to China. This pattern may reflect a growing global recognition of the importance of diabetic foot monitoring. The geographical diversity could indicate increasing international awareness; however, it may be influencing the adoption and development of these technologies [71]. Furthermore, our findings indicate that the majority of the included studies were clinical trials. RCTs ensure the production of reliable and robust data, which is essential for regulatory approvals, clinical guideline development, and public health policy decisions [72].

Since 2019, growing international research—primarily RCTs—reflects renewed interest and robust evidence supporting the adoption of wearable technologies for patients with DFUs. The prevalence of RCTs in this review may reflect an emphasis on generating high-quality evidence to support the use of wearable technologies for diabetic foot monitoring.

Although many of the 23 reviewed studies were RCTs—which strengthens their internal validity—this design does not automatically address 2 critical methodological limitations, including sample size and follow-up duration. The mean sample size across the included studies was only 26 participants, and many studies relied on single-session laboratory tests without any follow-up. This pattern indicates that most studies in this field were underpowered for developing generalizable prediction models, increasing the risk of overfitting and reducing external validity. Furthermore, the lack of follow-up means that dynamic preulcerative changes cannot be captured. Consequently, even within RCT designs, most studies provide only a biomechanical snapshot rather than a valid prognostic assessment, a limitation that some of the included studies explicitly acknowledged [28,31].

### Technical and Clinical Features of Wearable Systems

Sensor placement strategies across studies exhibited considerable heterogeneity, with common locations including the metatarsal heads, hallux, heel, midfoot, and arch (medial and lateral). These placements align with established biomechanical principles, as they enable the capture of temperature, pressure distribution, load transfer, and gait characteristics [73].

The multiple sensor locations on the foot are essential for effective detection and prediction of DFUs, as highlighted by multiple studies in this review. This strategic placement enables comprehensive monitoring of the key areas where ulcers are most likely to develop, improving early identification of risk and timely intervention. Jones et al [74] indicate that integrating parameters such as temperature, pressure, shear stress, and humidity across anatomically important sites yields richer data for prognosis. Many current models focusing on single parameters demonstrate limited predictive specificity (<50%), underscoring the need for multipoint and multisensor arrays precisely positioned to capture the foot microclimate comprehensively [74]. Although higher sensor density may improve spatial resolution, it remains unclear from the included studies whether this translates into better clinical prediction of DFUs, as no included study directly compared different sensor densities within the same population.

Despite these biomechanical rationales, the present review identified considerable heterogeneity in sensor placement protocols across the 23 included studies. This heterogeneity represents a key methodological limitation emerging from the reviewed studies. While the lack of a universal standardized protocol allows flexibility to address different research questions, it substantially complicates direct comparisons between studies and limits the feasibility of meta-analytic synthesis. Future research should therefore work toward establishing consensus-based guidelines for sensor placement to improve comparability, reproducibility, and clinical translatability across studies.

Also, a variety of foot monitoring systems were designed based on clinical and research requirements to address various needs [75]. Smart insoles appeared to be the most commonly developed solution, possibly due to their effectiveness. Socks and boot-based monitoring systems are other devices that may offer better support and stable sensor placement at high-risk anatomical sites [32,76]. The remaining studies either did not clearly specify the type of system or mainly worked with basic sensors and bandages. The range of application types shows the need for flexible solutions that can fit different users, such as people walking normally and engaging in sports, patients in clinics, or those needing home monitoring. Future research should continue to improve these wearable systems, making them more comfortable, easier to use, and better at collecting reliable data to help in real-world health care settings [29].

The reviewed studies consistently emphasize the importance of participant clinical history related to neuropathy and foot ulceration, particularly focusing on individuals with active DFUs or due to the high risk of recurrence. This participant characteristic is crucial as it identifies populations at high risk for ulcer recurrence and serious complications. Variation in participant selection across studies mirrors differing research goals. Studies aiming for early detection of risk factors or initial neuropathy may enroll broader diabetic populations without active ulcers to develop screening tools [77]. In contrast, studies evaluating monitoring technologies or treatment effectiveness often focus on patients with active or healed ulcers to assess prevention of recurrence and ulcer healing outcomes [78].

The number of sensors used across the 23 reviewed studies varied considerably, ranging from single-sensor systems to arrays of up to 42 sensors. Configurations included 5, 6, 8, 16, 24, and 42 sensors, depending on study objectives and measurement complexity. Studies requiring detailed localized pressure and temperature data tended to use higher sensor densities, whereas those focusing on broader gait parameters used fewer sensors for practicality.

External reviews reported that increased sensor density enhances spatial resolution, potentially boosting the precision of pressure and movement monitoring. Different sensor approaches for diabetic foot monitoring discuss trade-offs between sensor density, imaging modalities, and integration with AI tools for remote management. The sensor type and data resolution must align with application contexts and technical constraints [79]. Multifaceted wearable systems use a limited number of sensors embedded in insoles to capture clinically relevant pressure and temperature data while balancing wearability and adherence in daily life [80]. Reviews of plantar pressure and temperature sensing technologies confirm that high-density mats provide detailed foot surface mapping but face limitations for use in real-world ambulatory settings, where moderate sensor counts suffice and improve user compliance [81,82].

In this review, studies with higher sensor counts (eg, 42 sensors) were primarily technical in nature and did not report direct clinical outcomes such as ulcer prevention or healing. In contrast, studies using 5‐6 sensors (typically combining temperature and pressure) more frequently reported preventive outcomes. This pattern suggests that increasing the number of sensors does not necessarily translate into improved clinical outcomes, and there may be an optimal threshold beyond which additional technical complexity adds little clinical value.

Furthermore, no included study directly compared different sensor densities within the same population to determine whether higher spatial resolution improves clinical prediction of DFUs. The included studies also did not provide data on the trade-off between sensor density and patient adherence or comfort. Based on the available evidence, moderate sensor configurations (eg, 5‐8 sensors) may be sufficient for routine clinical use, but this hypothesis requires direct testing in future research.

In terms of sensing technologies used for plantar pressure monitoring, three main types of sensors are commonly used: piezoelectric, piezoresistive, and capacitive. Each offers distinct advantages in terms of sensitivity, durability, and energy efficiency. Despite the technological diversity available, most of the studies included in this review did not report the type of sensor technology used in their devices and typically name the commercial system.

This lack of detail may limit the ability to evaluate device performance, compare outcomes across studies, or draw conclusions about the relative effectiveness of different sensor types in predicting DFUs. Capacitive and resistive sensors dominate recent in-shoe pressure measurement, but no current system fully meets all ideal criteria—like accuracy over multiple load cycles, comfort, wireless communication, and affordability—highlighting ongoing challenges in sensor development. Furthermore, variability in measurement outcomes across devices complicates the assessment of their relative efficacy, underscoring the need for transparency in reporting sensor technologies [83,84].

Variation in communication methods reflects trade-offs between power consumption, data transfer rates, and connectivity stability. BLE has increasingly become the protocol of choice in numerous Internet of Things (IoT) devices, which commonly implement lightweight authentication and encryption methods to ensure secure communication [85]. The preference for wireless communication underscores the need for mobility and real-time data acquisition in gait and pressure analysis. Wi-Fi, though less frequently mentioned, offers advantages in terms of data transfer speed and network connectivity, making it a viable option for applications requiring continuous high-throughput data transmission. The problem is that Wi-Fi could be vulnerable to cyber-attacks. Wireless electronic sensors have the potential to reduce health care expenses by allowing physicians to remotely track key physiological information [86].

Wearable systems for diabetic foot monitoring use diverse sensor placements and technologies to capture critical biomechanical and physiological data. Multipoint sensor arrays improve early detection of DFUs by monitoring pressure, temperature, and other parameters in high-risk areas. Smart insoles and wireless communication, particularly via BLE, enhance usability and real-time remote monitoring capabilities. Despite these advancements, challenges remain regarding sensor accuracy, wearability, and the transparent reporting of technologies. Future improvements should target user comfort, data reliability, and integration with advanced analytics for better clinical outcomes.

### Key Metrics and Clinical Outcomes

Participant selection across the reviewed studies mainly focused on the importance of diabetic foot monitoring. Most studies included participants with a history of diabetes, either as the main group or as part of a mixed sample. Including people with diabetes highlights the practical use of sensor-based monitoring systems for the early detection and prevention of DFUs.

The evidence shows that most wearable or insole-based pressure sensor studies include patients with diabetes, reflecting a practical research approach targeting those who will most benefit from early detection of increased plantar pressure or temperature changes [87]. Large RCTs assessing the effectiveness of these monitoring systems also recruit diabetic cohorts predominantly to maximize clinical relevance and event detection [88].

Temperature and pressure are reported as key parameters in reviewed studies. The evidence also indicates that temperature and pressure are important early indicators, highlighting the significant role these 2 factors play in predicting DFUs. External evidence suggests temperature is a sensitive early warning sign, though models relying on a single parameter rarely exceed 50% specificity [74].

Patients with diabetes have higher plantar pressure than healthy individuals. Along with pressure and temperature, other factors like gait, balance, and skin humidity were monitored, as they influence DFU risk. Daily step counts provided insights into movement patterns and stress on the foot. Additional parameters—such as blood flow, shear stress, and galvanic skin response—were explored to offer a more comprehensive assessment of foot health and ulcer risk. Additional parameters such as gait, balance, humidity, blood flow, and shear stress were explored in a subset of studies [78,83]. Building on this, wearable sensors also improve clinical outcomes and patient care in diabetic foot management. Wearable sensors for DFUs in patients with diabetes provide a broad spectrum of clinical benefits, including early detection, prevention, improved healing, patient empowerment, remote monitoring, functional improvements, and cost-effectiveness. These outcomes support the integration of wearable technology into routine diabetic foot care to enhance patient outcomes and optimize health care resource use. While a few studies did not report direct clinical outcomes, the overall evidence strongly supports the integration of wearable sensor technology into routine DFU management to improve clinical outcomes.

Based on the included studies, wearable sensors demonstrated clinical benefits, including early detection, prevention, improved healing, patient empowerment, remote monitoring, functional improvements, and cost-effectiveness. External studies have also reported reduced neuropathic pain [89]; decreased acute care use, including hospitalizations and emergency visits, in a substantial proportion of studies [90]; and improved mental health outcomes [91], although these involved different device types and populations.

However, 3 important distinctions should be noted: (1) the external studies used different device types (eg, nerve stimulation devices, telehealth programs) rather than the in-shoe pressure/temperature sensors reviewed here; (2) the external studies often had larger sample sizes (eg, n=550 in [89]) compared to the mean of 26 participants in the present review; and (3) the external studies focused on outcomes such as pain and mental health, whereas the present review found that most included studies focused on biomechanical parameters (pressure, temperature) rather than direct patient-reported outcomes. Therefore, direct extrapolation of these external findings to wearable in-shoe monitoring systems for DFU prevention should be made with caution.

Also, these technologies provide comprehensive foot health assessment by integrating multiple physiological measures, advancing personalized care. Wearable devices also demonstrate significant clinical benefits, including neuropathic pain reduction, improved mobility, and decreased ulcer incidence. Remote patient monitoring and telehealth further enhance clinical outcomes and mental health by enabling timely, accessible support.

Overall, these related studies support and extend the initial findings by suggesting that wearable sensors may have potential benefits in the prevention and management of DFUs.

The integration of wearable monitoring devices in diabetic foot care has shown substantial promise in improving early detection, prevention, and management of foot complications. Wearable technologies have emerged as promising tools for enhancing foot health monitoring in individuals at risk and those who had DFUs previously.

This review has several limitations that should be considered when interpreting the findings. First, there was heterogeneity across the included studies in terms of study design, sample size, patient populations, device types, and outcome measures, which limited the ability to directly compare results or draw generalizable conclusions.

Second, many studies lacked comprehensive technical reporting, including details on sensor calibration, validation against gold standards, and data processing methods. This variability in reporting limits the reproducibility and comparability of findings across studies and, in some instances, necessitated reliance on external evidence to contextualize or support certain technical interpretations.

Finally, few studies reported strong clinical outcomes, such as ulcer occurrence, recurrence, infection, or amputation. Most studies relied on indirect measures, such as pressure or temperature, which may not fully represent real clinical benefit.

### Future Directions in Wearable Technology

The integration of AI and multimodal sensing represents a promising direction for the evolution of wearable technologies in DFU management. Multimodal sensing combines parameters such as plantar pressure, temperature, and shear stress. Emerging devices, including smart insoles, sensor-embedded socks, and external foot sensors, enable continuous monitoring of multiple physiological and biomechanical parameters, such as pressure, temperature, humidity, blood flow, gait, and balance. This shift toward data-driven, multiparameter systems may enhance predictive performance and improve clinical trust, addressing key limitations associated with single-parameter monitoring approaches.

### Clinical Implications and Patient-Centered Care

By capturing diverse data points, wearable technologies offer a comprehensive and multifactorial approach to assessing DFU risk. Their integration into routine diabetic care has the potential to support real-time monitoring and personalized feedback, enabling earlier detection of abnormalities and timely intervention.

This technology-driven approach may enhance patient empowerment and self-management, leading to improved adherence and clinical outcomes. Furthermore, continuous data collection can support more informed clinical decision-making and facilitate the development of personalized care strategies tailored to individual patient needs.

### Conclusion

Finally, few studies reported robust clinical outcomes, such as ulcer occurrence, recurrence, infection, or amputation. Most studies relied on indirect measures, such as pressure or temperature, which may not fully reflect real clinical effectiveness. Future research should focus on developing standardized reporting frameworks for wearable technologies to enable more consistent and meaningful comparisons across studies. There is also a need for pragmatic, real-world trials that include adherence-based interventions to better understand long-term outcomes and real-world performance. In addition, further high-quality studies are needed to evaluate the clinical benefits of these technologies and clarify their role within existing care pathways. If supported by stronger evidence, integrating wearable technologies into routine diabetic foot care may help improve adoption and patient outcomes.

## Supplementary material

10.2196/84463 Multimedia Appendix 1 Quality assessment, interrater agreement, and clinical outcomes of the included studies.

10.2196/84463 Checklist 1 PRISMA checklist.

## Abbreviations

BLE: Bluetooth low energy
DFU: diabetic foot ulcer
MMAT: Mixed Methods Appraisal Tool
NHS: National Health Service
PICO: Population, Intervention, Comparison, and Outcome
PRISMA: Preferred Reporting Items for Systematic Reviews and Meta-Analyses
RCT: randomized controlled trial
SID: Scientific Information Database
