# Supplementary material for: Wearable Technologies for Diabetic Foot Ulcer Monitoring and Risk Prediction: Systematic Review
Source: JMIR Diabetes. 2026 Jul 13;11:e84463. doi: 10.2196/84463 (PMC13361614; doi:10.2196/84463)
Supplement: Multimedia Appendix 1 [file diabetes-v11-e84463-s001.docx]

**Table S1.** Quality Assessment of Included Studies.

| **Quantitative randomised controlled trials** | | | | | | **Quantitative nonrandomised** | | | | |
| --- | --- | --- | --- | --- | --- | --- | --- | --- | --- | --- |
| Study | Appropriate  randomisation | Comparable  groups at  baseline | Complete  outcome  data | Assessors  blinded to the  intervention | Participants’  adherence  to the  intervention | Participants  representative  of the target  population | Appropriate  measurements  for the outcome  and intervention | Complete  outcome  data | Confounders  accounted for  in the design  and analysis | Administered  intervention |
| Hochlenert et al., 2025 [28] | Y | Y | Y | N | Y |  |  |  |  |  |
| Bulathsinghala et al., 2024 [29] |  |  |  |  |  | N | N | Y | N | Y |
| Cay et al., 2024 [30] | Y | N | Y | N | Y |  |  |  |  |  |
| Hu et al., 2024 [31] | Y | Y | Y | N | Y |  |  |  |  |  |
| Park et al., 2023 [32] |  |  |  |  |  | Y | Y | Y | N | Y |
| Tang et al., 2023 [33] |  |  |  |  |  | N | Y | Y | Cannot tell | Y |
| Gupta et al., 2023 [22] |  |  |  |  |  | Y | Y | Y | Cannot tell | Y |
| Armstrong et al., 2023 [34] |  |  |  |  |  | Y | Y | Y | N | Y |
| Khandakar et al., 2022 [3] |  |  |  |  |  | N | Y | Y | N | Y |
| Reyzelman et al., 2022 [35] |  |  |  |  |  | Y | Y | Y | N | Y |
| Scholten et al., 2022 [36] |  |  |  |  |  | Y | Y | Y | N | Y |
| Samarentsis et al., 2022 [37] |  |  |  |  |  | N | Y | Y | N | N |
| Beach et al., 2021 [38] | Y | Y | Y | N | Y |  |  |  |  |  |
| Du et al., 2021 [39] | Y | Y | Y | N | Y |  |  |  |  |  |
| González et al., 2021 [19] | Y | Y | Y | N | Y |  |  |  |  |  |
| Abbott et al., 2019 [40] | Y | Y | Y | Y | Y |  |  |  |  |  |
| Huchegowda et al., 2019 [41] |  |  |  |  |  | Y | N | N | N | Y |
| Ming et al., 2019 [20] | Y | Y | Y | N | Y |  |  |  |  |  |
| Reyzelman et al., 2018 [42] |  |  |  |  |  | Y | Y | Y | N | Y |
| Zhou et al., 2018 [43] |  |  |  |  |  | Y | Y | Y | Y | Y |
| Coates et al., 2016 [44] |  |  |  |  |  | Y | Y | Y | Y | Y |
| Grewal et al., 2015 [45] | Y | Y | Y | Y | Y |  |  |  |  |  |
| Wrobel et al., 2014 [46] |  |  |  |  |  | Y | Y | Y | N | Y |

Abbreviations: N, no; Y, yes.

**Table S2.** Cohen’s kappa Results.

| **No.** | **Paper** | **Reviewer 1** | **Reviewer 2** |
| --- | --- | --- | --- |
| 1 | Towards a Remote Patient Monitoring Platform for Comprehensive Risk Evaluations for People with Diabetic Foot Ulcers | Include | Include |
| 2 | Wearable laser Doppler flowmetry for non-invasive assessment of diabetic foot microcirculation: methodological considerations and clinical implications | Include | Include |
| 3 | A Polyester–Nylon Blend Plantar Pressure Sensing Insole for Person With Diabetes | Exclude | Exclude |
| 4 | Temperature Sensing Insoles for Diabetic Foot Ulcer Diagnostics | Exclude | Exclude |
| 5 | Intelligent plantar pressure offloading for the prevention of diabetic foot ulcers and amputations | Exclude | Exclude |
| 6 | Smart Offloading Boot System for Remote Patient Monitoring: Toward Adherence Reinforcement and Proper Physical Activity Prescription for Diabetic  Foot Ulcer Patients | Include | Include |
| 7 | A stretchable wireless wearable bioelectronic system for  multiplexed monitoring and combination treatment of  infected chronic wounds | Exclude | Exclude |
| 8 | A Wearable Insole System to Measure Plantar Pressure and Shear for People with Diabetes | Include | Include |
| 9 | Diabot: Development of a Diabetic Foot Pressure Tracking Device | Include | Include |
| 10 | Sensor-Assisted Wound Therapy in  Plantar Diabetic Foot Ulcer Treatment:  A Randomized Clinical Trial | Include | Include |
| 11 | A single arm prospective feasibility study evaluating wound closure with a unique wearable device that provides intermittent plantar compression and offloading in the treatment of non‐healing diabetic foot ulcers | Include | Include |
| 12 | Design and Implementation of a Smart Insole System to Measure Plantar Pressure and Temperature | Include | Include |
| 13 | An Evaluation of Real-world Smart Sock–Based Temperature Monitoring Data as a Physiological Indicator of Early Diabetic Foot Injury: Case-Control Study | Include | Include |
| 14 | Utilization of a Smart Sock for the Remote Monitoring of Patients With Peripheral Neuropathy: Cross-sectional Study of a Real-world Registry | Include | Include |
| 15 | A simple flexible printed capacitive pressure sensor for chronic wound monitoring | Exclude | Exclude |
| 16 | A3D-Printed Capacitive Smart Insole for Plantar  Pressure Monitoring | Include | Exclude |
| 17 | Wearable medical apparatus to  monitor temperature and pulsatile blood-flow signal on forefoot to  predict diabetic foot ulcers | Include | Include |
| 18 | Monitoring of Dynamic Plantar Foot Temperatures in Diabetes with Personalised 3D-Printed Wearables | Include | Include |
| 19 | Remote Diabetic Foot Temperature Monitoring  for Early Detection of Diabetic Foot Ulcers: A  Cost-Effectiveness Analysis | Exclude | Exclude |
| 20 | Modeling, Fabrication and Integration of Wearable Smart Sensors in a Monitoring Platform for Diabetic Patients | Exclude | Exclude |
| 21 | The Feasibility and Effectiveness of Wearable Sensor Technology in the Management of Elderly Diabetics with Foot Ulcer Remission: A Proof-Of Concept Pilot Study with Six Cases | Include | Include |
| 22 | The Use of Infrared Thermography to Develop and Assess a Wearable Sock and Monitor Foot Temperature in Diabetic Subjects | Include | Include |
| 23 | Podiatrist-Delivered Health Coaching to Facilitate the Use of a Smart Insole to Support Foot Health Monitoring in People with Diabetes-Related Peripheral Neuropathy | Exclude | Exclude |
| 24 | Clinical performance evaluation of a  newly developed sock for people with  diabetes | Exclude | Exclude |
| 25 | Use of a Remote Temperature Monitoring Mat for the Early Identification of Foot Ulcers | Exclude | Include |
| 26 | Remote Temperature Monitoring  in Patients With Visual Impairment  Due to Diabetes Mellitus: A Proposed  Improvement to Current Standard of  Care for Prevention of Diabetic Foot  Ulcers | Exclude | Include |
| 27 | Sensock: 3D Foot Reconstruction with Flexible Sensors | Exclude | Exclude |
| 28 | Innovative intelligent insole system reduces diabetic foot  ulcer recurrence at plantar sites: a prospective, randomised, proof-of-concept study | Include | Include |
| 29 | Integrative studies to design and validate wearable footwear among neuropathic patients | Include | Include |
| 30 | Study protocol for a randomised controlled  trial to test for preventive effects of  diabetic foot ulceration by telemedicine  that includes sensor-equipped insoles  combined with photo documentation | Include | Include |
| 31 | Development of Low Frequency (20-100 kHz) Clinically Viable Ultrasound Applicator for Chronic Wound Treatment | Exclude | Exclude |
| 32 | Continuous Temperature-Monitoring Socks for Home Use in Patients With Diabetes: Observational Study | Include | Include |
| 33 | Hemodialysis Impact on Motor Function beyond  Aging and Diabetes—Objectively Assessing Gait and  Balance by Wearable Technology | Include | Include |
| 34 | Wearable Multimodal Skin Sensing for the Diabetic Foot | Include | Include |
| 35 | Battery-Free Smart Sock for Abnormal Relative  Plantar Pressure Monitoring | Exclude | Exclude |
| 36 | Sensor-Based Interactive Balance Training with  Visual Joint Movement Feedback for Improving  Postural Stability in Diabetics with Peripheral Neuropathy: A Randomised Controlled Trial | Include | Include |
| 37 | New methods for evaluating physical and thermal comfort properties of orthotic materials used in insoles for patients with diabetes | Exclude | Exclude |
| 38 | Smart Diabetic Socks: Embedded device for diabetic foot prevention | Exclude | Exclude |
| 39 | A Novel Shear Reduction Insole Effect  on the Thermal Response to Walking  Stress, Balance, and Gait for Diabetic  Neuropathy | Include | Include |
| 40 | A Miniaturized, Battery-Free, Wireless Wound Monitor That Predicts Wound Closure Rate Early | Exclude | Exclude |
| 41 | Wearable device for iontophoretic treatment and monitoring of pressure ulcers: Proof-of-concept | Exclude | Exclude |
| 42 | Investigations on postural stability and spatiotemporal parameters of human gait using developed wearable smart insole | Exclude | Exclude |
| 43 | Design of a wireless and fully flexible insole using a highly sensitive pressure sensor for gait event detection | Exclude | Exclude |
| 44 | Relationship between skin temperature monitoring with Smart Socks and plantar pressure distribution: A pilot study | Exclude | Exclude |
| 45 | Flexible wound healing system for pro-regeneration, temperature monitoring and infection early warning | Exclude | Exclude |
| 46 | A wearable, self-sustainable, and wireless plantar pressure and temperature monitoring system for foot ulceration prognosis and rehabilitation | Exclude | Exclude |

**Table S3.** Results of clinical outcomes of studies.

| **No.** | **Study** | **Clinical outcomes** |
| --- | --- | --- |
| 1 | Hochlenert et al., 2025 [28] | Ulcer faster healing, preventing ulcer deterioration, patient-centric care, reduce pain, and improve quality of life. |
| 2 | Bulathsinghala et al., 2024 [29] | Early detection ulcers, reducing the need for frequent hospital visits, cost-effective, and timely intervention. |
| 3 | Cay et al., 2024 [30] | Identify patients at high risk of poor wound healing, supports personalised treatment plans, allowing targeted interventions. Moreover, early identification of barriers to healing, reduced the need for frequent in-person visits, and patient engagement. |
| 4 | Hu et al., 2024 [31] | Early detection of microcirculation dysfunction, potential for preventive interventions, suitable for routine clinical use, and support patient self-monitoring. |
| 5 | Park et al., 2023 [32] | Improves balance stability, real-time tracking of offloading adherence, and allows proactive intervention for non-adherence. |
| 6 | Tang et al., 2023 [33] | Enabling earlier intervention and prevention strategies, does not increase plantar pressure, and is sensitive enough to detect clinically relevant changes in pressure and shear. |
| 7 | Armstrong et al., 2023 [34] | A high rate of wound closure, accelerated healing times, significant wound area reduction, and decreased pain, all without serious adverse events. |
| 8 | Khandakar et al., 2022 [3] | Early detection of diabetic foot complications at-home monitoring for preventive care, cost-effectiveness, and provides detailed gait cycle analysis. |
| 9 | Reyzelman et al., 2022 [35] | Early detection, preventive care, remote monitoring, and personalised treatment. |
| 10 | Scholten et al., 2022 [36] | No direct clinical outcomes. |
| 11 | Samarentsis et al., 2022 [37] | Early diagnosis, rehabilitation monitoring, personalised care, and cost-effectiveness. |
| 12 | Beach et al., 2021 [38] | Earlier identification and prevention of DFUs. |
| 13 | Du et al., 2021 [39] | Prevention of ulcer recurrence, improvement in gait and balance, early risk identification, and supporting individualised care and follow-up. |
| 14 | Torreblanca  González et al.,2021 [19] | Early detection of foot complications, detect pre-ulcerative inflammation, prevention of DFUs and amputations, and long-term monitoring without disrupting daily activities. |
| 15 | Abbott et al., 2019 [40] | Ulcer recurrence reduction, longer ulcer-free survival, less increase in callus severity, reduce sustained pressure, and empowerment of patients to self-manage foot health. |
| 16 | Huchegowda et al., 2019 [41] | Reduction of plantar pressure, pain and symptom relief, potential for ulcer prevention, improved safety and comfort, and support for long-term management. |
| 17 | Ming et al., 2019 [20] | It is protocol. |
| 18 | Reyzelman et al., 2018 [42] | Early detection of complications, patient adherence and comfort, potential for preventive care, and consistency with clinical observations. |
| 19 | Zhou et al., 2018 [43] | Early detection of motor function deterioration. |
| 20 | Coates et al., 2016 [44] | No direct clinical outcomes. |
| 21 | Grewal et al., 2015 [45] | Improved mental health, improved postural stability, improved functional mobility, and compensates for proprioceptive deficits in diabetic neuropathy patients. |
| 22 | Wrobel et al., 2014 [46] | Reduced thermal responses to walking  improvement in gait initiation  preventing DFUs. |

## REFERENCES:

3. Khandakar A, Mahmud S, Chowdhury MEH, et al. Design and implementation of a smart insole system to measure plantar pressure and temperature. Sensors (Basel). Oct 7, 2022;22(19):7599. [doi: 10.3390/s22197599] [Medline: 36236697]

19. Torreblanca González J, Gómez-Martín B, Hernández Encinas A, Martín-Vaquero J, Queiruga-Dios A, Martínez-Nova A. The use of infrared thermography to develop and assess a wearable sock and monitor foot temperature in diabetic subjects. Sensors (Basel). Mar 5, 2021;21(5):1821. [doi: 10.3390/s21051821] [Medline: 33807804]

20. Ming A, Walter I, Alhajjar A, Leuckert M, Mertens PR. Study protocol for a randomized controlled trial to test for preventive effects of diabetic foot ulceration by telemedicine that includes sensor-equipped insoles combined with photo documentation. Trials. Aug 22, 2019;20(1):521. [doi: 10.1186/s13063-019-3623-x] [Medline: 31439007]

22. Gupta S, Jayaraman R, Sidhu S, et al. Diabot: development of a diabetic foot pressure tracking device. J. 2023;6(1):32-47. [doi: 10.3390/j6010003]

23. Srass H, Ead JK, Armstrong DG. Adherence and the diabetic foot: high tech meets high touch? Sensors (Basel). Aug 3, 2023;23(15):6898. [doi: 10.3390/s23156898] [Medline: 37571682]

24. Srivastava T, Thiagarajan G, Alon US, et al. Role of biomechanical forces in hyperfiltration-mediated glomerular injury in congenital anomalies of the kidney and urinary tract. Nephrol Dial Transplant. May 1, 2017;32(5):759-765. [doi: 10.1093/ndt/gfw430] [Medline: 28339567]

25. Page MJ, McKenzie JE, Bossuyt PM, et al. The PRISMA 2020 statement: an updated guideline for reporting systematic reviews. Int J Surg. Apr 2021;88:105906. [doi: 10.1016/j.ijsu.2021.105906] [Medline: 33789826]

26. Cohen J. A coefficient of agreement for nominal scales. Educ Psychol Meas. Apr 1960;20(1):37-46. [doi: 10.1177/001316446002000104]

27. Pace R, Pluye P, Bartlett G, et al. Testing the reliability and efficiency of the pilot mixed methods appraisal tool (MMAT) for systematic mixed studies review. Int J Nurs Stud. Jan 2012;49(1):47-53. [doi: 10.1016/j.ijnurstu.2011.07.002] [Medline: 21835406]

28. Hochlenert D, Bogoclu C, Cremanns K, et al. Sensor-assisted wound therapy in plantar diabetic foot ulcer treatment: a randomized clinical trial. J Diabetes Sci Technol. May 2025;19(3):692-698. [doi: 10.1177/19322968231213095] [Medline: 38006228]

29. Bulathsinghala RL, Wijeyaratne SM, Fernando S, Jayawardana TSS, Senadhipathi Mudiyanselage VUI, Kankanamalage SLS. Wearable medical apparatus to monitor temperature and pulsatile-blood-flow signal on forefoot to predict diabetic foot ulcers. Res J Text Apparel. Apr 10, 2024;28(2):169-184. [doi: 10.1108/RJTA-11-2021-0135]

30. Cay G, Finco MG, Garcia J, McNitt-Gray JL, Armstrong DG, Najafi B. Towards a remote patient monitoring platform for comprehensive risk evaluations for people with diabetic foot ulcers. Sensors (Basel). May 8, 2024;24(10):2979. [doi: 10.3390/s24102979] [Medline: 38793835]

31. Hu XX, Xing XM, Zhang ZM, et al. Wearable laser doppler flowmetry for non-invasive assessment of diabetic foot microcirculation: methodological considerations and clinical implications. J Biomed Opt. Jun 2024;29(6):065001. [doi: 10.1117/1.JBO.29.6.065001] [Medline: 38737791]

32. Park C, Mishra R, Vigano D, et al. Smart offloading boot system for remote patient monitoring: toward adherence reinforcement and proper physical activity prescription for diabetic foot ulcer patients. J Diabetes Sci Technol. Jan 2023;17(1):42-51. [doi: 10.1177/19322968211070850] [Medline: 35048739]

33. Tang J, Bader DL, Moser D, et al. A wearable insole system to measure plantar pressure and shear for people with diabetes. Sensors (Basel). Mar 15, 2023;23(6):3126. [doi: 10.3390/s23063126] [Medline: 36991838]

34. Armstrong DG, Orgill DP, Glat PM, et al. A single arm prospective feasibility study evaluating wound closure with a unique wearable device that provides intermittent plantar compression and offloading in the treatment of non-healing diabetic foot ulcers. Int Wound J. Mar 2023;20(3):853-860. [doi: 10.1111/iwj.13932] [Medline: 36054243]

35. Reyzelman AM, Shih CD, Tovmassian G, et al. An evaluation of real-world smart sock-based temperature monitoring data as a physiological indicator of early diabetic foot injury: case-control study. JMIR Form Res. Apr 1, 2022;6(4):e31870. [doi: 10.2196/31870] [Medline: 35363148]

36. Scholten HJ, Shih CD, Ma R, Malhotra K, Reyzelman AM. Utilization of a smart sock for the remote monitoring of patients with peripheral neuropathy: cross-sectional study of a real-world registry. JMIR Form Res. Mar 1, 2022;6(3):e32934. [doi: 10.2196/32934] [Medline: 35230248]

37. Samarentsis AG, Makris G, Spinthaki S, Christodoulakis G, Tsiknakis M, Pantazis AK. A 3D-printed capacitive smart insole for plantar pressure monitoring. Sensors (Basel). Dec 12, 2022;22(24):9725. [doi: 10.3390/s22249725] [Medline: 36560095]

38. Beach C, Cooper G, Weightman A, Hodson-Tole EF, Reeves ND, Casson AJ. Monitoring of dynamic plantar foot temperatures in diabetes with personalised 3D-printed wearables. Sensors (Basel). Mar 2, 2021;21(5):1717. [doi: 10.3390/s21051717] [Medline: 33801346]

39. Du C, Wang H, Chen H, et al. The feasibility and effectiveness of wearable sensor technology in the management of elderly diabetics with foot ulcer remission: a proof-of-concept pilot study with six cases. Gerontology. 2021;67(4):493-502. [doi: 10.1159/000513729] [Medline: 33657570]

40. Abbott CA, Chatwin KE, Foden P, et al. Innovative intelligent insole system reduces diabetic foot ulcer recurrence at plantar sites: a prospective, randomised, proof-of-concept study. Lancet Digit Health. Oct 2019;1(6):e308-e318. [doi: 10.1016/S2589-7500(19)30128-1] [Medline: 33323253]

41. Huchegowda R, Shruti A, Amarendra S, Shraddha T, Huchegowda C. Integrative studies to design and validate wearable footwear among neuropathic patients. Diabetes Metab Syndr. May 2019;13(3):2075-2079. [doi: 10.1016/j.dsx.2019.03.038]

42. Reyzelman AM, Koelewyn K, Murphy M, et al. Continuous temperature-monitoring socks for home use in patients with diabetes: observational study. J Med Internet Res. Dec 17, 2018;20(12):e12460. [doi: 10.2196/12460] [Medline: 30559091]

43. Zhou H, Al-Ali F, Rahemi H, et al. Hemodialysis impact on motor function beyond aging and diabetes-objectively assessing gait and balance by wearable technology. Sensors (Basel). Nov 14, 2018;18(11):3939. [doi: 10.3390/s18113939] [Medline: 30441843]

44. Coates J, Chipperfield A, Clough G. Wearable multimodal skin sensing for the diabetic foot. Electronics (Basel). 2016;5(3):45. [doi: 10.3390/electronics5030045]

45. Grewal GS, Schwenk M, Lee-Eng J, et al. Sensor-based interactive balance training with visual joint movement feedback for improving postural stability in diabetics with peripheral neuropathy: a randomized controlled trial. Gerontology. 2015;61(6):567-574. [doi: 10.1159/000371846] [Medline: 25721132]

46. Wrobel JS, Ammanath P, Le T, et al. A novel shear reduction insole effect on the thermal response to walking stress, balance, and gait. J Diabetes Sci Technol. Nov 2014;8(6):1151-1156. [doi: 10.1177/1932296814546528] [Medline: 25107709]
